# History of occupational accidents and near misses and sleepiness in Italian professional drivers: a cross-sectional study

**DOI:** 10.3389/fpubh.2025.1658465

**Published:** 2025-07-17

**Authors:** Lucia Palandri, Elena Righi, Patrizia Melloni, Fabriziomaria Gobba, Federico Ricci, Alberto Modenese

**Affiliations:** ^1^Department of Biomedical, Metabolic and Neural Sciences, University of Modena and Reggio Emilia, Modena, Italy; ^2^Clinical and Experimental Medicine PhD Program, University of Modena and Reggio Emilia, Modena, Italy; ^3^Studio Specialistico di Medicina del Lavoro, Poviglio, Italy

**Keywords:** work accidents, near miss, professional drivers, Epworth Sleepiness Scale, sleep

## Abstract

**Background:**

Sleep difficulties among professional drivers are a major concern for health and safety as they can be associated with increased tiredness while driving and can be related to an increased risk of accidents. Several lifestyle-related risk factors can have an important impact on sleep. This study aims to investigate whether a history of work accidents or near misses is associated with abnormal sleepiness among a sample of Italian professional drivers, considering individual and occupational factors that may influence this relationship.

**Methods:**

From 2018 to 2022, we conducted a cross-sectional study recruiting professional drivers attending mandatory health and safety training courses in accordance with Italian legislation. Participants completed a six-section questionnaire including personal information, sleep-related difficulties, occupational data, habits, history of accidents and near misses, and the Epworth Sleepiness Scale (ESS). We used multiple logistic regression models to calculate odds ratios (ORs) and 95% confidence intervals (CIs) for abnormal sleepiness.

**Results:**

The sample comprised 884 workers, predominantly male (99%), with mean age 49.4 years. Our study showed associations between several risk factors and excessive sleepiness. Consuming alcohol showed an increased probability of higher ESS scores. Similarly, snoring and having a history of occupational accidents or near misses showed increased probability of abnormal sleepiness.

**Conclusion:**

This study suggests a potential association between a history of near misses and abnormal sleepiness among professional drivers. The observed relationship underscores the need to address underlying sleep disorders, such as undiagnosed sleep apnea, which may contribute to excessive sleepiness and compromised safety. Drivers with a history of work accidents or near misses should be considered for screening programs targeting abnormal sleepiness, as these events may serve as early warning signs. Educational interventions to raise awareness and promote better sleep hygiene among this at-risk group could play a crucial role in improving both occupational health and road safety. Despite limitations inherent to self-reported data, our findings offer valuable insights for targeted prevention strategies.

## 1 Introduction

Sleep problems among professional drivers represent a major concern for the health and safety as they can be associated with an increased tiredness while driving, with a reduction of the attention and lengthening of reaction times (1, 2). These factors can determine an increased risk of accidents for the drivers as well as for the general public in the urban and extra-urban traffic (3–5). Several lifestyle-style related risk factors can have an important impact on sleep quality, such as age, overweight and obesity, smoke, alcohol consumption (6, 7).

Sleep quality may also be affected by several disease as cardiovascular, respiratory, metabolic, psychiatric and neurological diseases (8–11).

Professional drivers face significant occupational risk factors that predispose them to sleep disorders. These include prolonged exposure to road traffic, characterized by extended working hours that lead to cumulative job stress and potential disruption of circadian rhythms. The nature of work shifts, particularly night shifts and early morning or nocturnal work schedules, further exacerbates the likelihood of experiencing sleep-related problems. Irregular and demanding work patterns can substantially compromise the quality and duration of sleep, potentially increasing the risk of fatigue-related health and safety issues in this occupational group (12, 13).

One of the main tools applied within the occupational health surveillance programs of professional drivers in order to monitor the possible presence of pathologic sleepiness is the validated questionnaire named Epworth Sleepiness Scale (ESS) (14), which allows for a quantitative evaluation for potential drowsiness. This is a self-administered 8-items questionnaire where drivers are asked to rate their likelihood of falling asleep during various activities, based on a four-points Likert scale from 0 (extremely unlikely) to 4 (very likely). The total ESS score can range from 0 to 24: the higher the score, the higher the daily sleepiness of the workers, with values possibly related to abnormal conditions above the score of 8.

Considering these premises, the aim of this study is to evaluate potential associations between sleepiness and reported work accidents or near misses in a sample of Italian professional drivers, taking into account individual factors possibly moderating these relationships. The goal is to assess whether a history of work accidents and near misses could serve as an indicator of underdiagnosed sleepiness.

## 2 Methods

### 2.1 Study design and setting

In the period 2018–2022 we carried out an cross-sectional study recruiting all professional drivers participating to a series of mandatory occupational health and safety training courses based on the current Italian legislation (legislative decree n° 81 of the year 2008). To recruit participants, 29 in person training courses were used, all organized in a driving school in Lombardy, with participants arriving from the provinces of Mantua and Reggio Emilia as well. Each course lasted for 36 h with 7 h specifically dedicated to workers’ health and safety and held by an occupational physician (PM). During this part of the training, prior to talking about night apneas and sleeping disorders, subjects were asked to fill in a self-administered questionnaire. All participants (*n* = 1,285) were given complete information regarding the study project, informed that data collection was on a voluntary basis, and that they were free to withdraw from the study at any time. Eight hundred and eighty-four participants filled in the questionnaires, giving their consent to participate in the study.

### 2.2 Questionnaire description

The self-administered questionnaire used was composed of 6 different sections. The first 5 sections asked general questions on personal information (section 1), sleeping difficulties (section 2), occupational data (section 3), voluptuary habits (section 4) and information on accidents and near misses while driving (section 5). The sixth included the ESS questionnaire.

Section 1 investigated personal data such as age, gender, height, weight, educational level, marital status and number of children. Section 2 asked for difficulties in falling asleep, sudden awakening while sleeping, early awakening in the morning, insomnia, sleep satisfaction and ever use of sedatives/hypnotics based on a 4-points scale (never, seldom, sometimes, often). Other items were yes/no questions, investigating snoring, sleep apnea, excessive daily tiredness and hypertension. For the analysis, we transformed answers based on a 4-points scale to dichotomous variables, e.g., for sleep satisfaction we compared the positive response of those often satisfied with their sleep (satisfaction = yes) with those reporting relevant unsatisfaction (never, seldom and sometimes = no). The same categorization was applied for insomnia (“have you ever suffered from insomnia?”). Conversely, for the investigation of the use of sedative-hypnotics the question was “have you ever taken drugs to favor your sleep?”: in this case we separated those answering “never,” representing the group of “no sedatives-hypnotics” from the other respondents, categorized under the “yes” response.

Section 3 focused occupational data, enquiring about the usual working hours, the performance of long international travels, the type of activity as employee or self-employer and the job as courier.

Section 4 enquired about smoking and alcohol habits: we considered the questions “how many alcohol you take on a daily basis?” and we analyzed those answering “0 alcohol” as the “no alcohol group” vs. those reporting some quantities as the “yes” group; for smoking the question is “how many cigarettes do you smoke on a daily basis?” and also in this case we analyzed those indicating 0 cigarettes as the no smokers group vs. the others.

Section 5 investigated accidents and near miss history. Within this section we specifically analyzed the items “In the last 3 years have you had accidents at work while driving?” and “In the last 3 years have you had near miss accidents at work while driving?,” with the answers formulated based on a yes/no choice.

Finally, section 6 included the ESS scale, described above in the introduction paragraph. As the questionnaire was self-reported and administered for prevention purposes, we adopted the most precautionary interpretation of the scale, based on the original paper of Johns 1991 (14), where the normal controls are defined with a mean score of 5.9 ± 2.2, and accordingly we considered subjects with no sleepiness problems or with an average normal sleepiness those with scores ≤8, while an abnormal sleepiness for respondents scoring 9 or higher.

### 2.3 Statistical analysis

First, we performed descriptive statistics of the answers obtained with the questionnaire for the specific variables considered using the software SPSS version 29.0.1.0 for Windows and calculating absolute and relative frequencies for categorical variables and mean and Standard Deviation (SD) for continuous variables as data were normally distributed as assessed by the Shapiro–Wilk normality test. We calculated descriptive statistics for the overall population and for subgroups based on the results of the ESS (normal/average sleepiness scores vs. abnormal scores) and we evaluated differences between groups by Student’s *T*-test and Pearson’s or Chi-square test.

We then performed univariate logistic regressions to calculate crude Odds Ratios (OR) and 95% Confidence Intervals (95%CI). Finally, we carried out a multiple logistic regression model to calculate the OR and 95% CI for abnormal sleepiness in relation to individual characteristics and risk factors.

## 3 Results

### 3.1 Description of the population

The sample was composed of 884 workers. The majority of responders (99%) were males, with a mean age of 49.4 years (SD 8.6). Considering the education level, only 2.8% of the sample had a university degree, while 58% had only a secondary school diploma or lower education level (Table 1).

**Table 1:** Characteristics of the study sample.

		Total number	Percentage
		884	100
Socio-demographic and work-related variables			
Age (range 27–74)	≤40	133	15.3
	>40	736	84.7
	Unknown	15	–
Gender	Males	871	99.0
	Females	9	1.0
	Unknown	4	–
Education level	Primary school or less	63	7.2
	Secondary school	442	50.7
	High school	342	39.3
	University or higher	24	2.8
	Unknown	13	–
Years worked as professional driver (range 0–51)	0	6	0.9
	1–5	53	7.7
	6–15	167	24.3
	16–30	293	42.6
	>30	169	24.6
	Unknown	196	–
Kilometers driven/year (range 5,000–300,000)	5–10,000	56	9.0
	11–50,000	171	27.4
	51–100,000	276	44.2
	101–200,000	116	18.6
	201–300,000	5	0.8
	Unknown	260	–
Job status	employee	644	76.0
	Self-employer	203	34.0
	Unknown	37	–
Courier	No	701	85.2
	Yes	122	14.8
	Unknown	61	–
Long international transports	No	795	93.9
	Yes	52	6.1
	Unknown	37	–
Health information			
BMI	<25	194	23.1
	25- < 30	434	51.6
	≥30	213	25.3
	Unknown	43	–
Smoke	No	618	70.7
	Yes	256	29.3
	Unknown	10	–
Alcohol consumption	No	559	63.7
	Yes	318	36.3
	Unknown	7	–
Hypertension	No	764	88.2
	Yes	102	11.8
	Unknown	18	–
Sleep quality			
Sleep satisfaction	Never	56	6.7
	Seldom	130	15.5
	Sometimes	250	29.7
	Often	405	48.2
	Unknown	43	–
Insomnia	Never	583	67.7
	Seldom	153	17.8
	Sometimes	108	12.5
	Often	17	2.0
	Unknown	23	–
Sleep apnea	No	766	88.6
	Yes	99	11.4
	Unknown	19	–
Difficulties to get asleep	Never	400	45.7
	Seldom	256	29.2
	Sometimes	193	22.0
	Often	27	3.1
	Unknown	8	–
Snoring	No	615	71.6
	Yes	244	28.4
	Unknown	25	–
Use of sedatives/hypnotics	No	803	92.3
	Yes	70	7.7
	Unknown	11	–
Sleepiness			
Epworth Sleepiness Scale score	ESS score = normal (≤6)	512	62.1
	ESS score = average sleepiness (7- ≤ 8)	143	17.4
	ESS score = abnormal sleepiness (≥9)	169	20.5
	Unknown	60	–
Accidents and near miss accidents			
History of occupational accidents	No	801	93.1
	Yes	59	6.9
	Unknown	24	–
History of near miss occupational accidents	No	777	93.6
	Yes	53	6.4
	Unknown	54	–

The number of years worked as a professional driver was highly variable, from 0 years (i.e., new employers) to highly experienced drivers who had already retired but still working part-time with more than 50 years of employment, with an average length of employment of 22.5 years (±11.6 SD). Workers reported driving for an average of 73,409.1 km/year (±43,756 SD), with a wide range from 5,000 km/year to 300,000. Thirty-four percent of the drivers were self-employed, and considering the characteristics of the activity, only 6.1% of them reported performing long international transport and only 14.8% reported to work as courier (Table 1).

Regarding health status, 12.4% of the workers reported hypertension, 29.3% were smokers, 36.3% reported at least a low quantity of alcohol consumption daily and 51.6% of the subjects were overweight, with 25.3% resulting obese (BMI ≥ 30).

Considering sleep habits and sleep quality, 75% of the workers reported to never, or only seldom, have difficulties in falling asleep and only 2.0% of the sample reported to often have insomnia episodes. Drivers reporting to have taken sedatives-hypnotics at least once in the past were 7.7 and 11.4% had at least one episode of sleep apnea. Globally, about a half of the subjects (48.2%) declared being often satisfied with their sleep quality, and 28.4% reported snoring while asleep. Data of the ESS was available for 93% of the subjects: the mean score resulted 5.7 (±3.7 SD), with the 17.4% of the subjects reporting a score of 7 or 8, indicating an average sleepiness, while the 20.5% scored 9 or higher, resulting in an abnormal sleepiness (Table 1).

Finally, regarding work accidents as drivers, as well as near missed injuries, information was available for 97.3% of the sample for accidents and for 93.9% for near miss: 6.9% of the drivers reported at least one job incident while driving during their work activity, while 6.4% referred at least a case of near missed work injury (Table 1).

### 3.2 Associations between abnormal sleepiness and the investigated variables of interest

Table 2 reports the risk (OR and 95% CI) of having abnormal sleepiness in relation to various individual conditions, including age, gender, education levels, BMI, voluptuary habits, hypertension, sleep problems, history of occupational accidents and history of near miss occupational accidents. No significant differences resulted when evaluating age, the education level, BMI and hypertension. Female drivers showed an increased crude OR of 1.6 (95% CI = 1.3–4.9) of having abnormal sleepiness. Similar positive associations resulted with alcohol consumption and smoke (Table 2).

**Table 2:** Demographic and health related information, sleep quality, and work-related accidents or near misses in subgroups of drivers reporting normal or abnormal sleepiness as measured by ESS.

		Normal sleep or average sleepiness (ESS score ≤ 8) 79.5% (*n* = 665)	Abnormal sleepiness (ESS score ≥9) 20.5% (*n* = 169)	Crude Odds ratio (95% CI)	*p*-value
Age (mean years)		49.4 (±8.6), 79.4% (*n* = 647)	49.5 (±8.4), 20.6% (*n* = 168)	1.00 (0.98–1.02) For 1 year increment	0.948
Age	<=40	82.0% (*n* = 105)	18.0% (*n* = 23)	ref	0.421
Categories	>40	78.9% (*n* = 542)	21.1% (*n* = 145)	1.22 (0.75–1.99)	
Gender	Males	79.8% (*n* = 649)	20.2% (*n* = 164)	ref.	0.018
	Females	44.4% (*n* = 4)	66.6% (*n* = 5)	4.95 (1.31–18.62)	
Education level	Primary school	86.5% (*n* = 45)	13.5% (*n* = 7)	ref.	
	Secondary school	78.6% (*n* = 327)	21.4% (*n* = 89)	1.75 (0.76–4.01)	0.187
	High School	79.6% (*n* = 258)	20.4% (*n* = 66)	1.65 (0.71–3.81)	0.246
	University degree	81.0% (*n* = 17)	19.0% (*n* = 4)	1.51 (0.39–5.83)	0.548
BMI	<25	79.2% (*n* = 141)	20.8% (*n* = 37)	ref.	0.970
	≥25	79.3% (*n* = 484)	20.7% (*n* = 126)	0.99 (0.66–1.50)	
Smoke	No	82.1% (*n* = 471)	17.9% (*n* = 103)	ref.	0.006
	Yes	73.1% (*n* = 177)	26.9% (*n* = 65)	1.68 (1.18–2.40)	
Alcohol consumption	No	82.8% (*n* = 428)	17.2% (*n* = 89)	ref.	0.002
	Yes	76.5% (*n* = 222)	23.5% (*n* = 80)	1.73 (1.23–2.44)	
Hypertension	No	78.9% (*n* = 563)	21.1% (*n* = 151)	ref.	0.218
	Yes	84.2% (*n* = 85)	15.8% (*n* = 16)	0.702 (0.40–1.23)	
Sleep satisfaction	No	76.6% (*n* = 315)	23.4% (*n* = 96)	ref.	0.089
	Yes	81.6% (*n* = 314)	18.4% (*n* = 71)	0.74 (0.53–1.05)	
Insomnia	No	80.6% (*n* = 558)	19.4% (*n* = 134)	ref.	0.030
	Yes	71.2% (*n* = 84)	28.4% (*n* = 34)	1.69 (1.09–2.62)	
Sedatives/hypnotics	No	80.3% (*n* = 607)	19.7% (*n* = 149)	ref.	0.025
	Yes	68.3% (*n* = 43)	31.7% (*n* = 20)	1.90 (1.08–3.32)	
Snoring	No	84.8% (*n* = 487)	15.2% (*n* = 87)	ref.	<0.001
	Yes	67.0% (*n* = 156)	33.0% (*n* = 77)	2.76 (1.93–3.94)	
History of occupational accidents	No	80.4% (*n* = 604)	19.6% (*n* = 147)	ref.	0.015
	Yes	66.7% (*n* = 38)	33.3% (*n* = 19)	2.05 (1.15–3.67)	
History of Near miss occupational accidents	No	82.0% (*n* = 597)	18.0% (*n* = 131)	ref.	<0.001
	Yes	58.8% (*n* = 30)	41.2% (*n* = 21)	3.22 (1.76–5.89)	

Considering sleep problems, only sleep satisfaction did not result associated with the results of high ESS values. For workers with insomnia an increased likelihood of having abnormal sleepiness was observed (OR = 1.7; 95% CI = 1.1–2.6). For those who reported an assumption of sedatives-hypnotics the OR was 1.9 (95% CI = 1.1–3.3), while for the professional drivers who reported snoring at night the OR increased to 2.8 (95% CI = 1.9–3.9) (Table 2).

Finally, the crude association between the reporting of work-related accidents while driving in the previous 3 years and the presence of abnormal sleepiness resulted in an OR of 2.1 (95% CI = 1.2–3.7). For near miss occupational accidents, the association resulted stronger, with an OR of 3.2 (95% CI = 1.8–5.9), indicating a three-fold increased likelihood of having abnormal sleeping values if the driver had a near miss event in the previous three (Table 2).

In Table 3 we present the results of the multiple logistic regression model evaluating associations with abnormal sleepiness and drivers’ characteristics or risk factors as well as the history of occupational accidents or near misses.

**Table 3:** Multiple logistic regression: adjusted associations (OR and 95% CI) between abnormal sleepiness and related risk factors.

		Adjusted Odds ratio for abnormal sleepiness (95% CI)	*p*-value
Age		0.99 (0.97–1.02)	0.503
Gender	Males	ref.	
	Females	5.38 (0.99–29.33)	0.052
BMI	<25	ref.	
	≥25	0.94 (0.59–1.52)	0.810
Smoke	No	ref.	
	Yes	1.31 (0.86–1.99)	0.209
Alcohol consumption	No	ref.	
	Yes	1.46 (0.97–2.18)	0.068
Insomnia	No	ref.	
	Yes	1.61 (0.93–2.77)	0.087
Sedatives/hypnotics	No	ref.	
	Yes	1.04 (0.49–2.18)	0.922
Snoring	No	ref.	
	Yes	2.73 (1.83–4.06)	<0.001
History of occupational accidents	No	ref.	
	Yes	1.86 (0.92–3.78)	0.085
History of near miss occupational accidents	No	ref.	
	Yes	2.97 (1.48–5.98)	0.002

When considering personal characteristics and voluptuary habits, positive associations with abnormal sleepiness were found for gender with an adjusted odds ratio of 5.38 (95%CI: 0.99–29.33) and alcohol consumption, 1.46 (0.97–2.18).

Results showed how drivers who snored or had insomnia had a higher probability of having abnormal sleepiness values, respectively 2.73 (1.83–4.06) and 1.61 (0.93–2.77).

Finally, when considering reported work-related accidents, workers with a history of occupational related accidents showed a probability of 1.86 (0.92–3.78) times of having abnormal sleepiness, which is even higher when considering near miss occupational accidents, 2.97 (1.48–5.98).

## 4 Discussion

This study aimed to investigate whether a history of work-related accidents and near misses could be associated with abnormal sleepiness among professional drivers, and to explore whether such events might serve as indicators of underdiagnosed sleep disorders. Drawing on data from a large sample of Italian professional drivers, our findings support this hypothesis: drivers with a history of near misses or accidents were significantly more likely to report excessive daytime sleepiness, as measured by the Epworth Sleepiness Scale (ESS), even after adjusting for potential confounders.

The association was particularly strong for drivers reporting near miss incidents, who demonstrated nearly three times the odds of experiencing abnormal sleepiness (adjusted OR = 2.97; 95% CI: 1.48–5.98). These findings highlight near misses not only as precursors to potentially serious accidents, but also as potential red flags for underlying sleep-related impairments that may otherwise go unrecognized. This suggests that a history of workplace safety incidents, including near misses and accidents, could be considered as potential indicators for identifying drivers who may benefit from sleep health screening and tailored educational programs. Moreover, these findings suggest that role of education and continuous training as well as communication skills of healthcare professionals could be beneficial in early detection and management of sleep disorders (15–17).

In addition to accident history, other personal and behavioral characteristics were linked to increased sleepiness. Female drivers, those consuming alcohol, individuals reporting snoring or insomnia, and those using sedative-hypnotics all had elevated odds of reporting abnormal sleepiness. These findings reinforce the multifactorial nature of sleep-related risk in professional drivers and point to modifiable factors that could be addressed through occupational health strategies.

This data are in accordance to other findings reported in other scientific literature, from different parts of the world: the study of Souza et al. (18) in a group of Brazilian truck drivers found that the scores obtained at the ESS were higher in the group of drivers that had had accidents compared to those that had not (mean score 8.2 ± 4.2 vs. 6.3 ± 4.1). In this study the percentage of drivers who reported snoring was significantly lower compared to our study (11.1% vs. 28.4%), and this may be important as also snoring resulted significantly associated with excessive sleepiness according to our results. Considering alcohol consumption, the Brazilian study indicated a prevalence of daily alcohol intake among professional drivers relevantly higher compared to our study (50.9% vs. 36.3%).

In an Iranian study (19), high scores in the ESS questionnaire in 556 occupational road drivers resulted in increased likelihood of having a history of road accidents (OR = 1.13; 95% CI: 1.07–1.23), and in this study the percentage of workers who reported to snore was lower (10.7%) compared to our study. A Korean study (20) on 143 train drivers reported that near miss events were associated with ESS scores higher or equal to 11 with an OR of 8.65 (CI 95% 1.02–73.42), adjusted for age, working years, shiftwork, obesity, smoking, binge drinking, regular exercise, and caffeine consumption, while in the model adjusted for sleep quality, insomnia, OSA, daytime sleepiness the OR resulted increased but not significantly when looking at the confidence intervals (OR = 4.48; 95% CI: 0.48–41.34).

Considering Europe, a Belgian study on 476 truck drivers found that the mean ESS score was 6.79 (SD 4.17) (12). In the multiple logistic regression analysis, low educational level (OR = 1.86), smoking (OR = 1.75), unrealistic work schedule (OR = 1.75) and risk for obstructive sleep apnea (OR = 2.97) were found to be independent correlates of daytime sleepiness. Similarly, we found a crude positive association between sleepiness and smoke in our study, with an OR of 1.7 (95% CI 1.2–2.4). On the other hand, the Belgian study did not specifically consider associations with accidents and near miss events, and the alcohol consumption did not result as associated to sleepiness, which differ from the results we obtained.

As a final remark, despite alignment with findings from comparable research, our study has several methodological limitations. The cross-sectional design precludes causal inference despite observed associations. Additionally, the reliance on self-reported data introduces potential response bias, with participants possibly underreporting health conditions, lifestyle behaviors or work related incidents. Nevertheless, the study’s substantial sample size (884 participants), implementation of validated ESS instrumentation facilitating literature comparison, and concordance of findings with existing evidence constitute the primary strengths of this investigation.

## 5 Conclusion

We conducted a cross-sectional study involving a large sample of professional drivers from northern Italy who participated in mandatory occupational health and safety training courses between 2018 and 2022. Our findings reveal that a history of occupational accidents and, more notably, near misses is significantly associated with increased daytime sleepiness as assessed by the Epworth Sleepiness Scale (ESS).

Among the predictors of excessive sleepiness, snoring emerged as the strongest factor, suggesting a high prevalence of potential undiagnosed sleep-disordered breathing in this population. Alcohol consumption was also significantly associated with abnormal ESS scores, highlighting the importance of considering modifiable lifestyle-related factors in occupational health evaluations. While an association with female gender was observed, this finding should be interpreted with caution due to the small number of female drivers in the sample and warrants further investigation in future research.

These results underscore the value of using prior safety incidents—especially near misses—as early warning signals for identifying drivers at risk of sleep-related impairment. Integrating sleep health screening and educational interventions into routine occupational health programs, and enhancing the training of healthcare professionals in recognizing sleep disorders, could help mitigate risks and promote safer driving practices among this high-risk workforce.

## Data Availability

The raw data supporting the conclusions of this article will be made available by the authors, without undue reservation.
